# The Predictivity of Serum Biochemical Markers in Acute Biliary Pancreatitis

**DOI:** 10.5402/2011/279607

**Published:** 2010-12-14

**Authors:** Bülent Güngör, Kasım Çağlayan, Cafer Polat, Deniz Şeren, Kenan Erzurumlu, Zafer Malazgirt

**Affiliations:** ^1^Department of Surgery, Faculty of Medicine, Ondokuz Mayıs University, 55139 Samsun, Turkey; ^2^Department of Surgery, Faculty of Medicine, Bozok University, Yozgat, Turkey

## Abstract

*Background and Aim*. There are no accurate methods of differentiating acute biliary pancreatitis. Obstructions of biliary ducts, idiopathic pancreatitis may be related with biliary origin which needs identification for acute treatment. We searched for the predictivity of biochemical markers in early acute biliary pancreatitis. *Patients and Methods*. Serum levels of AST (Aspartate Transaminase),ALT (Alanine Transaminase), ALP (Alkaline Phosphatase), GGT (Gamma Glutamyl Transferase), total bilirubin, direct bilirubin, LDH (Lactate Dehydrogenase), amylase, lipase, CRP (C-Reactive Protein) and WBC (White Blood Cell) were measured in 157 patients with acute pancreatitis. Biliary and nonbiliary pancreatitis were differentiated by Magnetic Resonance Cholangiopancreatography (MRCP), Endoscopic Retrograde Cholangiopancreatography (ERCP), Intraoperative Cholangiopancreatography (IOC). Cut-off points of admission biochemical markers with sensitivity, specifity, positive predictive value and negative predictive value were determined after identification of significant variables. Receiver Operator Curves were plotted for each biochemical marker. *Results*. Serum Alkaline Phosphatase, total bilirubin, direct bilirubin, amylase and lipase levels were significantly higher in biliary pancreatitis with a positive predictive value of 80.8%, 83.9%, 81.6%, 78.8%, 79.7%. *Conclusion*. Increased Alkaline Phosphatase,total bilirubin, direct bilirubin, amylase and lipase levels may be used in prediction of biliary pancreatitis.

## 1. Introduction

Acute pancreatitis is a disease that could result in systemic inflammatory response, sepsis, multiorgan failure, and death. The severity of the inflammation, hemorrhage, or necrosis of the pancreas, peripancreatic fluid collection, or abscess are closely related with prognosis [1–3]. Bile reflux into the common bile duct and obstruction of the Ampulla Vater by biliary sludge or stone are supposed to cause biliary pancreatitis [4]. The obstruction of the common bile duct and the pancreatic duct separately or together may effect the resolution of the pancreatitis. Also transient or permanent obstruction, acute or chronic obstruction, and type of obstruction (like tumor or stone, etc.) of these ducts are effective on the prognosis and determine the management. If the obstruction persists more than 48 hours, the complications increase [3, 5–7]. The stones in the distal common bile duct and the pancreatic duct could not be detected in all patients with biliary pancreatitis. We do not know if these are actually biliary pancreatitis or not. There are no simple and accurate means of differentiating acute biliary pancreatitis from other types of pancreatitis [8, 9]. We tried to find the biochemical predictive factors of biliary pancreatitis in order to determine the sequence of the diagnostic and therapeutic methods for cost- and time-effective purposes. 

## 2. Patients and Methods

A retrospective analysis of 157 patients with mild and severe acute pancreatitis admitted to Ondokuz Mayıs University, Faculty of Medicine, Department of Surgery between 1995–2010 was performed. The diagnosis of acute pancreatitis was established by increased hepatic enzymes, serum amylase level, and computerized tomography findings. The age, sex, and medical history of the patients were recorded. Serum levels of AST (aspartate transaminase), ALT (alanine transaminase), ALP (alkaline phosphatase), GGT (gamma-glutamyl transferase), total bilirubin, direct bilirubin, LDH (lactate dehydrogenase), amylase, lipase, CRP (C-reactive protein), and WBC (white blood cell) were measured. Results of magnetic resonance-cholangiopancreatography (MRCP), endoscopic-retrograde-cholangiopancreatography (ERCP), and the time of laparoscopic cholecystectomy were evaluated. We defined the predictive ability of admission biochemical markers in patients whose etiology of pancreatitis were determined as biliary by USG, MRCP, ERCP, and intraoperative cholangiography (IOC). Univariate analysis by unpaired student *t* test with two tail distribution was used for quantitative variables. Nonsignificant variables were eliminated from univariate analysis. Cut-off points of admission values of biochemical markers with sensitivity, specificity, positive predictive value, and negative predictive value were determined after the identification of significant variables. Receiver operator curves (ROC) were plotted for each biochemical marker. SPSS 15.0 version was used for the statistical analysis. 

## 3. Results

The analysis demonstrated that 112 (71.33%) of 157 patients underwent biliary evaluation, 78 by ERCP and 34 by MRCP. The mean age of the patients was 61; seventy-three patients were female, and 39 patients were male. Biliary evaluation occurred in first week of admission. Fourteen (41.17%) patients who underwent MRCP had common bile duct stones. The common bile duct stones were also determined on ERCP. Five (6.41%) ERCPs failed due to technical difficulties and duodenal diverticula, and these patients were operated. Sixty-two (79.48%) ERCPs were positive for common bile duct stones and 1 for biliary ascariasis. All these patients had sphincterotomy after biliary irrigation. Thirty-four patients underwent cholecystectomy within 15 days, and 58 underwent within 30 days. Mean time to laparoscopic cholecystectomy was 27 days (range 7–36). Five patients in whom ERCP was unsuccessful, underwent intraoperative cholangiography. In 3 of these patients, common bile duct stones were detected and converted to open surgery and choledochoduodenostomies were performed. 

**Figure 1:**
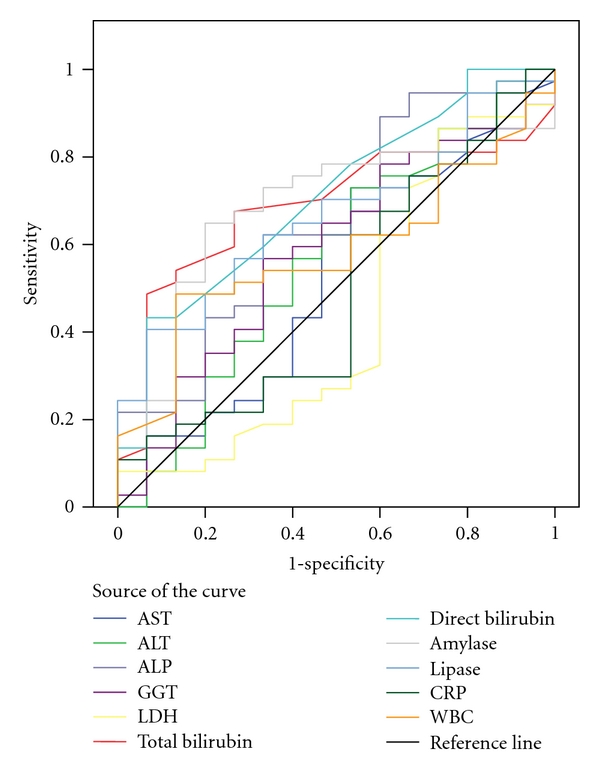
The ROC curve of all of the measured serum biochemical markers. Diagonal segments are produced by ties.

**Figure 2:**
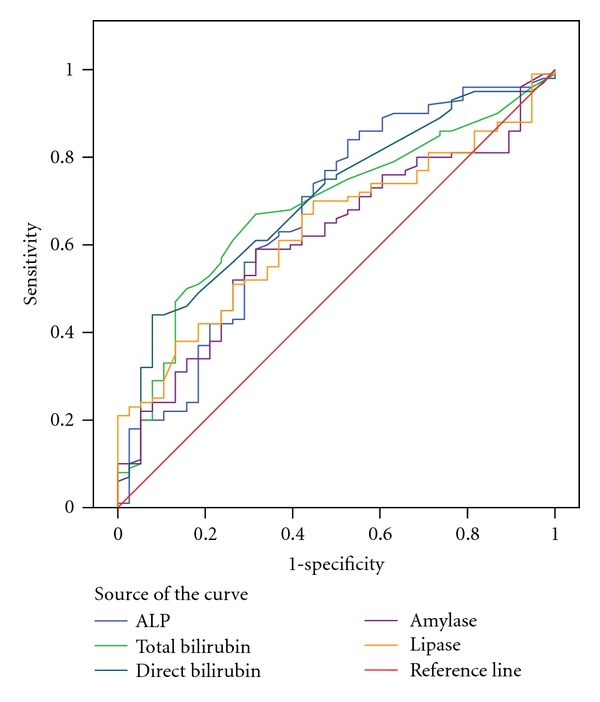
The ROC curve of the significantly increased biochemical markers in biliary pancreatitis. Diagonal segments are produced by ties.

 The biochemical markers whose values were significantly different between biliary and nonbiliary pancreatitis were demonstrated in Table 1. The Receiver Operator Curve (ROC) of all of the measured serum biochemical markers was demonstrated in Figure 1. The ROC curve of the significantly increased biochemical markers in biliary pancreatitis was demonstrated in Figure 2. 

## 4. Discussion

Morbidity and mortality of acute pancreatitis may be higher in biliary type. Distinguishing biliary pancreatitis from nonbiliary is important for the management like cholecystectomy, ERCP, and sphincterotomy. The period between the onset of symptoms and ERCP in biliary pancreatitis is controversial. Therefore, the early determination of biliary origin is extremely important. The accuracy of USG in detecting cholelithiasis and choledocholithiasis decreases during the attack of acute pancreatitis. The sensitivity of the USG is 70% in detecting gallstones [10]. The biochemical analysis revealed low predictive values for the etiology of pancreatitis. But most of the cases of idiopathic pancreatitis are now supposed to be biliary in origin due to occult biliary stones. Endoscopic ultrasonography (EUS) is helpful in detection of these occult stones, biliary sludge, or sand. Biliary sludge is known as echogenic, gravitating material composed of cholesterol crystals, calcium bilirubinate granules, and muco-glycoproteins [11, 12]. Three times increase in serum amylase level is diagnostic for acute pancreatitis. The specificity of amylase level is 95%, but the sensitivity is 61% similar to our result 60.6% (Table 1). The sensitivity of amylase decreases in alcoholic pancreatitis, hypertriglyceridemia. In alcoholic pancreatitis, the exocrine pancreatic insufficiency may occur secondary to chronic alcohol abuse [8, 9]. The sensitivity of amylase increased in biliary pancreatitis in our study as it was seen on ROC may be related with the sufficient exocrine function of the pancreas. More than two to three times increase in upper limit of serum amylase is used as a cut-off value. It was 970 IU/L in our study. The specificity of amylase decreases in intra-abdominal inflammations. In hypertriglyceridemia, sensitivity of amylase decreases due to competition of triglycerides with amylase assay. Serum lipase is derived mainly from pancreatic acinar cells. The serum lipase level rises 4–8 hours after the onset of symptoms, and peaks at the 24th hour of onset. It correlates closely with serum amylase levels and shows similar sensitivity and specifity [8]. We also found similar sensitivities and specificities for amylase and lipase. The pancreas is healthy in biliary pancreatitis different from hypertriglyceridemia-related and alcoholic pancreatitis; the pancreas is suddenly affected by a biliary sludge or stone. In alcoholic pancreatitis, amylase will not raise as high as in biliary pancreatitis. One fourth of the patients with alcoholic pancreatitis has normal levels of amylase. Lipase to amylase ratio of more than 3 may indicate alcoholic pancreatitis with a low sensitivity. Lipase increases 4 times more than amylase in chronic pancreatic insufficiency. The sensitivity of lipase differs from 55% to 100% in acute pancreatitis. We found its sensitivity as 61.2% and positive predictive value as 79.7% in acute biliary pancreatitis. When the amylase, total and direct bilirubin levels were measured in patients with initial pancreatitis after clearance by ERCP and in patients with post-ERCP pancreatitis, all the levels of these parameters decreased after relief of obstruction in biliary ducts. The average half-lives of amylase and lipase were longer in initial biliary pancreatitis, while the average peak values were higher in the post-ERCP pancreatitis. This shows the importance of the levels of amylase, lipase, and bilirubins in biliary pancreatitis and also the half-lives of amylase and lipase [13]. 

 The combination of EUS with biochemical findings may increase the diagnosis of biliary pancreatitis. The biochemical parameters suggesting biliary origin of pancreatitis may direct us to the endoscopic examination and treatment although there was no cholelithiasis determined in USG in acute pancreatitis. USG has a 25% false negative rate in detecting cholecystolithiasis. Because of low specificity of the biochemical markers, patients could undergo unnecessary ERCP examinations. In clinics where EUS is unavailable, biochemical parameters could be used in diagnosis for the early management. In populations where the incidence of biliary pancreatitis is high, sensitivity of the biochemical parameters may increase. 

 The bilirubin levels both total and direct had the highest specificity, positive, and negative predictive values among other biochemical laboratory measurements in our study. Of course the increased total bilirubin is related with the increase in the direct bilirubin. Like in obstructive jaundice, bilirubin levels (especially the direct bilirubin) were shown to be the most important markers of biliary pancreatitis. The cut-off values of bilirubins were very low (1 and 0.21 mg/dL). This shows that the pancreatitis may occur after very little obstruction of the common bile duct. The common channel theory, the dilated common bile duct more than 5 mm or the large angle between common bile duct and pancreatic duct may contribute to this effect. We could not detect the obstruction of the pancreatic duct or any other pathophysiological mechanism causing inflammation and lysis in the pancreas during the initial periods of development of pancreatitis. The little obstruction may cause a decreased drainage of the pancreatic secretions, or the bile or its pressure may cause or aggravate the pancreatitis or the obstruction in the pancreatic duct may be extremely large while little in bile duct. The imaging of the pancreatic duct, and the determination of the pancreatic and biliary secretions in the ampullary region, the pressure differences of the pancreatic and bile ducts, anatomy of the ducts should be defined to understand the mechanisms of biliary pancreatitis. 

 ALP, a significant parameter in biliary pancreatitis, also increases in obstructive jaundice. The significance of increase in ALP more than 246 U/L may indicate the obstruction in the ducts causing biliary pancreatitis. 

 We identified significant increase in five biochemical variables on admission which correlated with the biliary pancreatitis. These are ALP, total bilirubin, direct bilirubin, amylase, and lipase. In the literature, individual clinical parameters that correlate with biliary pancreatitis and biliary stones have been identified [5–7, 9]. Common bile duct size on ultrasound, ALP, GGT, total bilirubin, and direct bilirubin levels correlated with the presence of persistent choledocholithiasis. The age, bilirubin, ALP, ALT, and amylase levels were found significantly increased in biliary pancreatitis [14]. Lin et al. identified initial common bile duct size and AST level as significant predictors of choledocholithiasis in biliary pancreatitis [15]. Chang et al. showed total bilirubin, ALP, and ALT as significant variables [5]. Liu et al. found that age over 58 years, female gender, or ALT value > 150 U/L was associated with biliary etiology in pancreatitis [12]. The positive predictive value of ALT value > 150 U/L is 95%. Jaundice with increased ALT suggests gallstone etiology requiring ERCP [11, 12]. ALT or AST levels more than three times the upper limit of normal indicates gallstones as the cause of acute pancreatitis. However, the absence of elevated transaminases does not rule out gallstones. ALT has high specificity, but low sensitivity for gallstone pancreatitis. Sensitivities of elevated bilirubin and/or ALP levels in predicting common bile duct stones were reported as being paramount in attack of acute pancreatitis. Cohen et al. demonstrated rise in any serum biochemical marker within 24–48 hours of admission correlated with a persistent common bile duct stone in 31% of patients [6]. The biliary stones less than 2 mm in diameter, calcium bilirubinate crystals, cholesterolosis, biliary sludge, or sand may also cause biliary pancreatitis and elevate the biochemical markers. The majority of common bile duct stones pass into the duodenum within 48 hours. These cases could not always be diagnosed as biliary pancreatitis. They may be defined as idiopathic. Microlithiasis is said to be present in 40–80% of idiopathic pancreatitis [16]. ALP, total bilirubin, and direct bilirubin levels were known to increase in obstructive jaundice. So, the increase correlates with the biliary origin of pancreatitis. But interestingly, the serum amylase and lipase levels also increased significantly more than in nonbiliary pancreatitis. This may contribute to that acute biliary pancreatitis may result in more increase in pancreatic enzyme secretion or passage of these enzymes into the circulation when compared to nonbiliary pancreatitis. In practical purposes, when these five markers were elevated during admission to the hospital, the biliary evaluation should have priority not to delay in treatment. 

 CRP and leukocyte number did not differ between biliary and nonbiliary pancreatitis. They both are predictive in detecting the severity of pancreatitis rather than etiology. ALT, AST, and GGT levels did not also take place above the reference line of the ROC graphic in our study. This shows the less sensitivity of these individual parameters in our patients in distinguishing etiology. Clinical and radiological evaluation should complete the biochemical markers to find the exact cause of acute pancreatitis. 

 MRCP is a noninvasive procedure which could be the first diagnostic method when the suspicion of biliary pancreatitis occurred. It has 91% sensitivity and 98% specificity for detection of common bile duct stones [17]. Endoscopic ultrasonography would be useful in these patients in aspect of rapid diagnosis; furthermore, therapeutic intervention by ERCP could be applied on the same session if required [12, 18]. Negative MRCP and ERCP values varying from 40–80% in biliary pancreatitis make the necessity of improved reliability of biliary pancreatitis [10]. The limit of detection of the stones is 1 mm for MRCP, and stones smaller than 5 mm may be overlooked. Intraoperative cholangiography is also recommended in patients with cholecystolithiasis and suspicion of biliary pancreatitis that could not be diagnosed by MRCP, EUS, and ERCP [16, 19, 20]. As a conclusion, because of these high rates of negative MRCP and ERCP, biochemical parameters gain an important role in the selection of initial biliary evaluation for acute pancreatitis. 

## Figures and Tables

**Table 1:** The biochemical markers whose values were significantly different between biliary and nonbiliary pancreatitis.

	Cut-off value	Nonbiliary number	Biliary number	Sensitivity %	Specificity %	PPV %	NPV %
ALP	<246 U/L	23	38	62.4	60.5	80.8	37.7
	>246 U/L	15	63				
Total bilirubin	<1.0 mg/dL	29	42	65.4	67.4	83.9	42.9
	>1.0 mg/dL	11	62				
Direct bilirubin	<0.21 mg/dL	26	42	59.6	65.0	81.6	38.2
	>0.21 mg/dL	14	62				
Amylase	<970 IU/L	23	41	60.6	57.5	78.8	35.9
	>970 IU/L	17	63				
Lipase	<1400 IU/L	24	40	61.2	60.0	79.7	37.5
	>1400 IU/L	16	63				

ALP: alkaline phosphatase.

PPV: positive predictive value.

NPV: negative predictive value.

## References

[1] Wang GJ, Gao CF, Wei D, Wang C, Ding SQ (2009). Acute pancreatitis: etiology and common pathogenesis. *World Journal of Gastroenterology*.

[2] Carr-Locke DL (2003). Biliary pancreatitis. *Canadian Journal of Gastroenterology*.

[3] Johnson C, Lévy P (2010). Detection of gallstones in acute pancreatitis: when and how?. *Pancreatology*.

[4] Frossard JL, Steer ML, Pastor CM (2008). Acute pancreatitis. *The Lancet*.

[5] Chang L, Lo SK, Stabile BE, Lewis RJ, De Virgilio C (1998). Gallstone pancreatitis: a prospective study on the incidence of cholangitis and clinical predictors of retained common bile duct stones. *American Journal of Gastroenterology*.

[6] Cohen ME, Slezak L, Wells CK, Andersen DK, Topazian M (2001). Prediction of bile duct stones and complications in gallstone pancreatitis using early laboratory trends. *American Journal of Gastroenterology*.

[7] Roston AD, Jacobson IM (1997). Evaluation of the pattern of liver tests and yield of cholangiography in symptomatic choledocholithiasis: a prospective study. *Gastrointestinal Endoscopy*.

[8] Smotkin J, Tenner S (2002). Pancreatic and biliary disease: laboratory diagnostic tests in acute pancreatitis. *Journal of Clinical Gastroenterology*.

[9] Chan T, Yaghoubian A, Rosing D (2008). Total bilirubin is a useful predictor of persisting common bile duct stone in gallstone pancreatitis. *American Surgeon*.

[10] Moon JH, Cho YD, Cha SW (2005). The detection of bile duct stones in suspected biliary pancreatitis: comparison of MRCP, ERCP, and intraductal US. *American Journal of Gastroenterology*.

[11] Matull WR, Pereira SP, O’Donohue JW (2006). Biochemical markers of acute pancreatitis. *Journal of Clinical Pathology*.

[12] Liu CL, Fan ST, Lo CM (2005). Clinico-biochemical prediction of biliary cause of acute pancreatitis in the era of endoscopic ultrasonography. *Alimentary Pharmacology and Therapeutics*.

[13] Choi JH, Kang NL, Choi SD (2009). Serum enzyme half life can be a useful factor for follow-up management of biliary pancreatitis. *Clinical Biochemistry*.

[14] Davidson BR, Neoptolemos JP, Leese T, Carr-Locke DL (1988). Biochemical prediction of gallstones in acute pancreatitis: a prospective study of three systems. *British Journal of Surgery*.

[15] Lin G, Halevy A, Girtler O, Gold-Deutch R, Zisman A, Scapa E (1997). The role of endoscopic retrograde cholangiopancreatography in management of patients recovering from acute biliary pancreatitis in the laparoscopic era. *Surgical Endoscopy*.

[16] de Waele E, op de Beeck B, de Waele B, Delvaux G (2007). Magnetic resonance cholangiopancreatography in the preoperative assessment of patients with biliary pancreatitis. *Pancreatology*.

[17] Hallal AH, Amortegui JD, Jeroukhimov IM (2005). Magnetic resonance cholangiopancreatography accurately detects common bile duct stones in resolving gallstone pancreatitis. *Journal of the American College of Surgeons*.

[18] Chak A, Hawes RH, Cooper GS (1999). Prospective assessment of the utility of EUS in the evaluation of gallstone pancreatitis. *Gastrointestinal Endoscopy*.

[19] Draganov P, Forsmark CE (2005). “Idiopathic” pancreatitis. *Gastroenterology*.

[20] Garg PK, Tandon RK, Madan K (2007). Is biliary microlithiasis a significant cause of idiopathic recurrent acute pancreatitis? A long-term follow-up study. *Clinical Gastroenterology and Hepatology*.

